# Application of Solid Phase Extraction and High-Performance Liquid Chromatography with Fluorescence Detection to Analyze Bisphenol A Bis (2,3-Dihydroxypropyl) Ether (BADGE 2H_2_O), Bisphenol F (BPF), and Bisphenol E (BPE) in Human Urine Samples

**DOI:** 10.3390/ijerph181910307

**Published:** 2021-09-30

**Authors:** Tomasz Tuzimski, Szymon Szubartowski

**Affiliations:** 1 Department of Physical Chemistry, Medical University of Lublin, Chodźki 4a, 20-093 Lublin, Poland; szymon.szubartowski95@gmail.com; 2 Doctoral School of Medical University of Lublin, Medical University of Lublin, Chodźki 7, 20-093 Lublin, Poland

**Keywords:** bisphenols, bisphenol A bis (2,3-dihydroxypropyl) ether (BADGE 2H_2_O), human urine samples, fluorescence detector (FLD), dispersive solid phase extraction (d-SPE), solid phase extraction (SPE), Scherzo SM-C18

## Abstract

In this study, we propose a simple, cost-effective, and sensitive high-performance liquid chromatography method with fluorescence detection (HPLC-FLD) for the simultaneous determination of the three bisphenols (BPs): bisphenol A bis (2,3-dihydroxypropyl) ether (BADGE 2H_2_O), bisphenol F (BPF), and bisphenol E (BPE) in human urine samples. The dispersive solid phase extraction (d-SPE) coupled with solid phase extraction (SPE) procedure performed well for the analytes with recoveries in the range of 74.3–86.5% and relative standard deviations (RSD%) less than 10%. The limits of quantification (LOQs) for all investigated analytes were in the range of 11.42–22.35 ng mL^−1^. The method was validated at three concentration levels (1 × LOQ, 1.5 × LOQ, and 3 LOQ). During the bisphenols HPLC-FLD analysis, from 6 min a reinforcement (10 or 12) was used, therefore analytes might be identified in the small volume human urine samples. The results demonstrated clearly that the approach developed provides reliable, simple, and rapid quantification and identification of three bisphenols in a urine matrix and could be used for monitoring these analytes.

## 1. Introduction

Endocrine disruptors (EDs) are exogenous chemical substances that exert actions primarily through nuclear hormone receptors, including estrogen, androgen, progesterone, thyroid, and retinoid receptors, and consequently cause adverse health effects in an intact organism, its progeny, or (sub) populations. EDs have effects on male and female reproduction, breast development and cancer, prostate cancer, thyroid impairment, metabolism and obesity, and neurological, cardiovascular, and endocrine diseases [1,2].

Bisphenols are a widely used plastic compound with endocrine-disrupting properties that ubiquitously affect the endocrine system [2]. Bisphenol A (BPA), the most widely used bisphenol compound, is well known for having a negative effect on human reproductive health and has therefore been gradually eliminated from many commonly used products [3,4]. In particular, its use in baby bottles and toys has been prohibited by the EU [5]. Due to BPA toxicity, *European Food Safety Authority* (EFSA) evaluated scientific studies and set the maximum specific migration limit (SML) level to 0.05 milligrams per kilogram of food (mg/kg) in 2018, updating its previous level set in 2011 [6]. Regulations forced producers to introduce BPA analogues to the market, for example, bisphenols S (BPS), F (BPF), and B (BPB) that can be found in everyday-use products, including “BPA-free” labelled ones, and being suspected of similar toxicity to BPA. They can be found in environmental and biological samples and exhibit similar or even higher toxicity [7,8,9,10]. Nowadays, current evidence indicates that gametes are more sensitive to low doses of BPS, whereas the oocyte cytoskeleton, epigenetic code, and/or protein post-translational modifications are affected in a subtle way, depending on the biological impact of bisphenol [11,12]. Both BPS and BPF exert distinct biological effects on oocytes during the perinatal exposure window when the ovarian pool meiotic competence of oocytes is established [13]. Interestingly, individual bisphenols seem to differ in their molecular activity and result in different phenotypes in BPS- vs. BPF-exposed females [13]. That is why the EU put SML at 0.05 milligrams per kilogram of food for one of BPA analogue-BPS [14].

Humans are practically constantly exposed to the toxic effects of bisphenols, various metabolites of which can pass through inhalation, skin, or food to the human body [1]. The most common sources are various epoxy-based adhesives, plastic polycarbonate containers for food storage, and plastic films for food packaging. The situation is aggravated when the food is heated in dishes using microwave ovens because heating increases the extraction rate of BPs significantly [15,16]. Therefore, the majority of bisphenols enter the human body with food. The main route of their penetration into the human body is oral, followed by the respiratory and dermal tract [1].

Once in the body, it is rapidly absorbed from the gastrointestinal tract and undergoes a practically complete metabolism in the liver by conjugation with glucuronic acid [1,17]. The formed glucuronide is rapidly removed from the blood entering the urine [1,17]. 

Epoxy resin systems are important commercial formulations that are used widely in the construction protective coating of steel structures, such as bridges, storage tanks, and wind turbines, for their excellent adhesion to surfaces, durability, and anticorrosive properties. Epoxy resins used on steel structure coatings derive almost exclusively from **B**isphenol **A D**i**G**lycidyl **E**ther (BADGE) representing 75–95% of the total epoxide market [17,18,19].

Hydrolysis by epoxide hydrolase is the major biotransformation/detoxification pathway of epoxides, including BADGE, in humans, leading to three major metabolic byproducts: bisphenol A (2,3-dihydroxypropyl) glycidyl ether [BADGE H_2_O], bisphenol A bis (2,3-dihydroxypropyl) ether [BADGE 2H_2_O], and bisphenol A (3-chloro-2-hydroxypropyl) (2,3-dihydroxypropyl) ether [BADGE HCl H_2_O]. BADGE 2H_2_O is the major product, followed by BADGE HCl H_2_O and BADGE H_2_O. All three are excreted in urine as free metabolites as well as conjugates with sulphate and glucuronide [17,18].

Bello et al. [17] found that the BADGE 2H_2_O biomarker is the most predominant urinary hydrolysis biomarker of BADGE in studies of workers and provided high sensitivity (quantified in 100% of urine samples) and specificity (able to discriminate between various exposure scenarios and cross-shift changes). According to the study [17], the BADGE 2H_2_O is the most preferred biomarker for routine biomonitoring purposes and for establishing future urinary biological monitoring guidance values (BMGV).

Oxidation of BADGE to BADGE 2H_2_O by epoxide hydrolases appears to be the preferred metabolic pathway in humans and explains why the intermediate oxidation product BADGE H_2_O was found only in a small number of samples. BADGE H_2_O is hydrolyzed quickly to BADGE 2H_2_O by epoxide hydrolases, whereas further oxidation of BADGE 2H_2_O by monooxygenases to other oxidation byproducts proceeds more slowly, leading to accumulation of BADGE 2H_2_O in urine [17]. The second most abundant biomarker, BADGE HCl H_2_O, was present in urine in much lower concentrations, ~9 × lower than BADGE 2H_2_O. In light of Bella et al. [17] observations, establishing a reliable BMGV for BADGE 2H_2_O for occupational exposures will require additional background biomonitoring and health effect data.

Therefore, urine is the most suitable matrix for monitoring the content of such bisphenols. Their quantitative analysis in most cases is carried out using a variety of mass spectrometric techniques. Most of the described methods are based on the use of high-performance liquid chromatography coupled with mass spectrometry (HPLC-MS) or tandem mass spectrometry (HPLC-MS/MS) [20,21], as well as gas chromatography coupled with mass spectrometry (GC-MS) or tandem mass spectrometry (GS-MS/MS) [22,23,24,25]. 

A monitoring of six bisphenols and diethylstilbestrol in human urine samples was also described using the high-performance liquid chromatography coupled with a photodiode array detector (HPLC-PDA) [26].

Previous studies detected bisphenols in amniotic fluid, follicular fluid, placental tissue, sperm, cord blood, fetal serum, adipose tissue [27,28,29,30,31], and human breast milk samples [32,33,34,35]. 

Therefore, constant human biomonitoring requires the development of novel, effective, simple, not expensive, and highly sensitive methods of quantitative analysis of these xenobiotics in biological samples.

The developed and validated method was newly termed reversed phase high-performance thin-layer chromatography planar yeast ant-/agonistic androgen screen (RP-HPTLC-pYAAS bioassay) [36]. Results were also compared with the RP-HPTLC-*Aliivibrio fischeri* bioassay (applied on RP plates for the first time). As proof of concept, the transfer to another bioassay detecting estrogens (RP-HPTLC-pYES) was successfully demonstrated, analogously termed RP-HPTLC-pYAES bioassay detecting anti-/estrogens (exemplarily shown for evaluation of four pharmaceuticals used in breast cancer treatment) [36].

The validation of a procedure for the determination by GC-MS of three bisphenols (BPs), BPA, BPF, and bisphenol Z (BPZ), in seven human organs and tissues (kidney, liver, heart, lung, spleen, brain and abdominal brain, and abdominal fat) obtained from eight autopsies has been described [30].

In the present work, a low cost and sensitive method is proposed for quantitative analysis of selected bisphenols in urine samples. The procedure is especially dedicated to the identification and quantification of BADGE 2H_2_O which is the most preferred biomarker for routine biomonitoring purposes and for establishing future urinary BMGV [17].

To the best of our knowledge, this method is the first to combine the advantages of d-SPE/SPE as extraction techniques with HPLC-FLD. This could aid in the identification and quantification of bisphenol residues in human urine samples.

## 2. Materials and Methods

### 2.1. Chemicals and Reagents

The following standards used for the bisphenols under investigation were obtained from Sigma–Aldrich (Bellefonte, PA, USA): 3-[4-[2-[4-(2.3-Dihydroxypropoxy)phenyl]propan-2-yl]phenoxy]propane-1.2-diol (BADGE·2H_2_O, No. 1), bisphenol F (BPF, No. 2). bisphenol E (BPE, No. 3), and 4-Phenylphenol as internal standard (IS). The standard purity indicated by the manufacturers for all of the reference standards of bisphenols was ≥98.0%

### 2.2. Solvents and Mobile-Phase Solutions

LC-MS grade methanol (MeOH), gradient grade for liquid chromatography (LC) MeCN, and formic acid were obtained from E. Merck (Darmstadt, Germany), LC-MS grade water was purchased from Sigma–Aldrich (St. Louis, MO, USA). Deionized water (0.07–0.09 mS cm^−1^) was produced in our laboratory using a Hydrolab System (Gdańsk, Poland). All analytical equipment, including solvents and reagents, was checked for bisphenol contamination prior to analysis by HPLC-FLD. Individual stock standard solutions were prepared in methanol and stored in screw capped glass tubes in the refrigerator (+2 to +4 °C in the dark). A bisphenol standards mixture contained in all the analytes was prepared by combining suitable aliquots of each individual standard stock solution and diluting them with MeOH. The standard mixture was stored under the same conditions as individual stock standard solutions for up to 2 weeks. This mixture was used for calibration preparation as well as for fortification of the human urine samples. 

### 2.3. Apparatus and HPLC-FLD Conditions

An Agilent Technologies 1200 HPLC system with a Fluorescence Detector (Agilent Technologies 1260 FLD), a quaternary pump, and an autosampler that could thermostat samples were used for the LC analysis. Analytes were separated using a Scherzo SM-C18 150 × 4.6 mm column, with a 3-µm particle size (Agilent Technologies, Wilmington, DE, USA). The column was thermostated at 22 °C. The mobile phase consisted of 50 mM formic acid in water (component A) and 50 mM formic acid in MeCN (component B) in gradient elution: 0–10 min from 40% eluent B to 100% B; 10–14.5 min isocratic 100% B. The mobile phase flow rate was 0.4 mL/min. Then an autosampler (1260 Infinity II Vialsampler) was thermostated at 8 °C.

In order to elute interferences of the matrix, before the next step of human urine samples analysis, the isocratic elution with 100% B as the mobile phase was applied for 15 min with flow rate of 1 mL/min. Next, the isocratic elution with the initial conditions was performed before next analysis. 

FLD detection was carried out simultaneously at four different excitation wavelengths (225, 230, 235, and 240 nm). The optimal emission wavelength was set at 300 nm.

### 2.4. HPLC-FLD Analysis and Method Validation

A validation study was performed using spiked urine samples and included evaluation of the selectivity, linearity, limits of detection (LODs), LOQs, extraction recovery, process efficiency, and precision and accuracy. 

Quantitative analysis of bisphenols in urine samples was performed at the reinforcement equals 12, while the reinforcement 10 was used during determining the recovery value of analytes for samples spiked at 300 ng/mL. The reinforcements were appropriately matched to the value of the analytes’ signals.

#### 2.4.1. Selectivity 

The selectivity was evaluated by analyzing the urine samples from different sources to investigate the potential interferences with the signal of analytes. The extent of interferences originating from endogenous urine sample components at the specific retention time of each analyte was evaluated through a comparison of an average blank human urine matrix sample (collected from five men (ages from 25 to 27) and then mixed) with the spiked and average blank human urine matrix sample. HPLC analyses of bisphenols standards were repeated five times. The identification of bisphenols was accomplished on the basis of the retention times of the analytes. 

#### 2.4.2. Linearity

The linearity of the method was studied by spiking the average blank human urine matrix sample with suitable amounts of bisphenol standards. Samples were prepared according to d-SPE/SPE and determined by the HPLC-FLD method described in the Experimental Section 2.4. The solutions of the bisphenol standards were added to the average blank human urine matrix sample. The calibration curves for the LOD and LOQ values were constructed by analyzing bisphenol standards in MeOH at seven concentrations, ranking from 10 to 500 ng/mL using six replicates. The calibration curves were obtained by means of the least square method.

The LODs and LOQs obtained for bisphenols were calculated according to the formulas: LOD = 3.3 (SD/S) and LOQ = 10 (SD/S), where SD is the standard deviation of the response (peak area) and S is the slope of the calibration curve. HPLC analyses of bisphenols standards were repeated five times. 

The calibration curves for mLOQ values were constructed by analyzing spiked average blank human urine matrix sample at nine concentrations, over the range of 10–500 ng/mL using six replicates. The calibration curves were obtained by means of the least square method.

The identification of bisphenols was accomplished on the basis of the retention times of the analytes. 

### 2.5. Enzymatic Standard and Solutions

β-Glucuronidase from *Helix pomatia* type H1 was obtained from Sigma–Aldrich (St. Louis, MO, USA). The enzymatic solution was prepared weekly by dissolving the β-Glucuronidase purified powder in 1 M ammonium acetate (acetic acid was added to pH = 5) to obtain a solution of 3500 U/mL.

### 2.6. Preparation of Urine Samples before SPE

The conditions for the extraction of bisphenols from urine samples were optimized using the best, most optimal variant of sample preparation, i.e., to 500 µL of the urine sample was added 500 µL of a mixture of standard bisphenols of an appropriate concentration (as well as 4-Phenylphenol as an IS, and then also added: 150 µL of water, 200 µL of acetate buffer pH = 5, and 250 µL of β-Glucuronidase. Samples were incubated at 37 °C for 18 h.

### 2.7. SPE-Based Extraction Procedure

A Baker SPE 12G system with pump (No. N022.AN18) and Strata Phenyl (55 μm, 70 Å) 500 mg/6 mL SPE columns (Phenomenex, 8B-S006-HCH) were used. For SPE, each Strata Phenyl SPE column was conditioned with 5 mL of MeOH and 5 mL of water. After being loaded, the urine sample diluted to 100 mL (volume of urine sample ≤1.6 mL; flow rate, 10 mL/min; and pressure 75 mmHg), the elution of analytes was performed with 10 mL 1% formic acid (HCOOH) in n-heptane/MeOH 10/90 (*v/v*). Eluates were reconstituted in 300 µL of MeCN/H_2_O 30:70 (*v/v*) and then analyzed by HPLC-FLD.

During the quantification of bisphenols in urine samples, individual eluates were evaporated separately. After evaporating to dryness, they were connected and reconstituted in 300 µL MeCN: water 30:70 (*v/v*) (3 × 100 µL).

### 2.8. Calculation of Relative Standard Deviation Values (RSD) and Extraction Recovery

Accuracy in all cases was expressed as percentage recovery of the analyte using equation:

$$\mathrm{Recovery\%}=\frac{\mathrm{Average}\;\mathrm{analyte}\;\mathrm{concentration}\;\mathrm{found}\;\mathrm{in}\;\mathrm{the}\;\mathrm{spiked}\;\mathrm{urine}\;\mathrm{sample}}{\mathrm{Analyte}\;\mathrm{concentration}\;\mathrm{added}\;\mathrm{to}\;\mathrm{the}\;\mathrm{spiked}\;\mathrm{urine}\;\mathrm{sample}}\times 100\%$$

Precision was expressed as RSD% calculated as follows:

$$\mathrm{RSD\%}=\frac{\mathrm{Standard}\;\mathrm{deviation}\;\mathrm{of}\;\mathrm{the}\;\mathrm{recovery}\;\left(\%\right)}{\mathrm{Mean}\;\mathrm{recovery}\;\left(\%\right)}\times 100\%$$

Intra-laboratory reproducibility was studied for SPE procedure. 

(The use of 4-phenylphenol as an IS did not significantly increase the recovery values (up to 9%) for the quantified bisphenols in human urine samples (preliminary experiments)).

### 2.9. Dispersive Solid Phase (d-SPE) Salts

Single-packaged sorbents used to prepare the sets (their mixtures) used during the d-SPE stage, such as clean PSA and Z-Sep, were obtained from Sigma–Aldrich (Bellefonte, PA, USA).

### 2.10. The d-SPE Stage before SPE-Based Extraction Procedure 

Human urine samples were transferred to 15 mL falcon centrifuge tubes and spiked with an appropriate amount of a mixture of bisphenol standards and 2 mL of MeCN. Tubes were shaken vigorously for two minutes and centrifuged for 5 min three times (6000 rpm, 3480 rcf). 

The MeCN layer was transferred into 100 mL glass flask and diluted to 100 mL of deionized water to prepare sample for SPE clean-up step on Strata Phenyl SPE column (see Section 2.7).

### 2.11. Human Urine Sample Collection

Urine samples were obtained from volunteers living in Lublin: seventeen men aged 25 to 27, who declared that they did not take any medications, dietary supplements, etc. Urine from five men was mixed and used as an average human urine matrix sample (blank).

Twelve urine samples from 26-year-old men, who declared that they were not taking any medications, dietary supplements, etc., were tested for bisphenols present (Table 1).

All samples were collected in the glass bottles and immediately analyzed or frozen immediately at −23 °C until analysis. This study was approved by the ethics committee of the Medical University of Lublin, Poland (No. KE-0254/271/2018).

## 3. Results and Discussion

Dualde et al. [35] described the method for determination of BPA, BPF, BPS, and four parabens in human breast milk samples. The described procedure included an extraction and clean-up procedure based on the QuEChERS (Qu—quick, E—easy, Ch—cheap, E—effective, R—rugged, S—safe) methodology followed by liquid chromatography coupled to triple quadrupole mass spectrometry determination. The analytes were quantified in 10 milk samples in a range from <LOQ (limit of quantification) to 7.00 ng·mL^−1^ [35].

Tuzimski and Szubartowski [34] described a method for the determination of selected bisphenols in human breast milk samples by a dispersive solid phase extraction before solid phase extraction procedure (d-SPE/SPE) and high-performance liquid chromatography coupled with fluorescence detector (HPLC-FLD). In comparison to the results published earlier [33], the authors proposed a method, which has advantages, as follows [34]:

1. A 10–fold reduction in the sample volume (from 5 to 0.5 mL);
2. Optimization of the d-SPE/SPE technique for the majority of analyzed bisphenols;
3. Optimal recovery values obtained for all of the analytes in the range from 57 to 88% for seven bisphenols combined with a low matrix effect, ensuring the reliable identification and quantification of analytes;
4. The sample volume of 0.5 mL enabled to combine several milk samples from one woman allowing the identification and quantitation of the analytes in biological samples using a sensitive fluorescence detector (FLD);
5. Due to the use of HPLC-FLD it was possible to identify and quantify bisphenols in human milk samples.

Yi et al. [37] analyzed BPA with LC/MS/MS and HPLC/FLD in human breast milk and conducted a comparison of two methods in analyzed BPA levels. The LOQs were similar in the two methods, i.e., 1.8 and 1.3 ng/mL for the HPLC/FLD and LC/MS/MS assays, respectively [36]. In addition, the detection range of BPA was broader in the HPLC method than the LC/MS/MS method [37].

In this study, d-SPE clean up followed by SPE procedure were elaborated. A series of experiments were conducted applying HPLC-FLD system, which is less expensive than liquid chromatography coupled with mass spectrometry (LC-MS) or tandem mass spectrometry (LC-MS/MS). Bisphenol standards (see Table 2) were chromatographed in condition based on the previously published method applied for determination of selected bisphenols, after appropriate modification [33,34]. 

Targeted analytes were separated on a Scherzo SM-C18 column using a mobile phase of a simple composition (water and acetonitrile (MeCN), both acidified with 50 mM formic acid (HCOOH)). These conditions provide a suitable chromatographic performance in terms of selectivity and efficiency for the bisphenols under investigation (Table 3). All baseline resolved peaks of bisphenols were obtained and were selected for qualitative analysis of bisphenols in human urine samples. The HPLC-FLD method showed satisfactory accuracy and precision for the analysis of selected bisphenols in human urine samples. Next, the optimization of the clean-up step of the d-SPE method was performed. The amount of sorbents in the d-SPE stage have a key role in the result so these were optimized. For this purpose, two sorbents were tested, including the relatively new commercially available sorbents such as zirconium dioxide-based Z-Sep and PSA. Primary secondary amine (PSA) is a weak anion exchanger sorbent with the ability to remove sugars, organic acids, fatty acids, and polar pigments, while its chemical structure provides a high chelating effect. Z-Sep, Z-Sep+, and EMR-Lipid (enhanced matrix removal-lipid) removes fatty and more hydrophobic interferences of matrices [38,39,40,41,42,43]. Zirconium-based dispersive phases demonstrate the ability to extract more fatty non-polar interferences (e.g., lipids) and pigment than traditional PSA and octadecyl (C18) sorbents and, therefore, in many cases, greater analyte recovery and better reproducibility may be achieved [34].

The ratio of PSA to Z-SEP was chosen during optimization (on the basis of previous experiments [33,34]). Additionally, Z-Sep with PSA can remove some medicines, vitamins, and dietary supplements in urine. Lastly, two different extraction protocols were developed. In one procedure, the d-SPE conditions were applied before SPE for proper extraction purification. In the second procedure, the d-SPE stage was omitted and the SPE procedure was only applied. The experimental conditions were the same, but the amounts of Z-Sep and PSA were different before the recovery of the three spiked samples was determined. The best following approach regarding the purification step of the extracts was evaluated using 50 mg Z-Sep and 30 mg PSA during the d-SPE step; extracts were also tested without d-SPE purification. The average recoveries for studies of analytes were compared (for the best protocol). The results showed that 74–87% recovery was achieved for the bisphenols at 50 mg Z-Sep and 30 mg PSA. 

Conditions for the SPE experiment were kept the same. SPE was performed using Strata Phenyl SPE columns which can be applied especially even for hydrophobic analytes. In addition to hydrophobic interactions, more selective adsorption is possible by π–π interactions due to the electron density of the phenyl ring. Due to these characteristics of the Strata Phenyl SPE columns, the average recovery values of the analytes were satisfactory.

**Figure 1:**
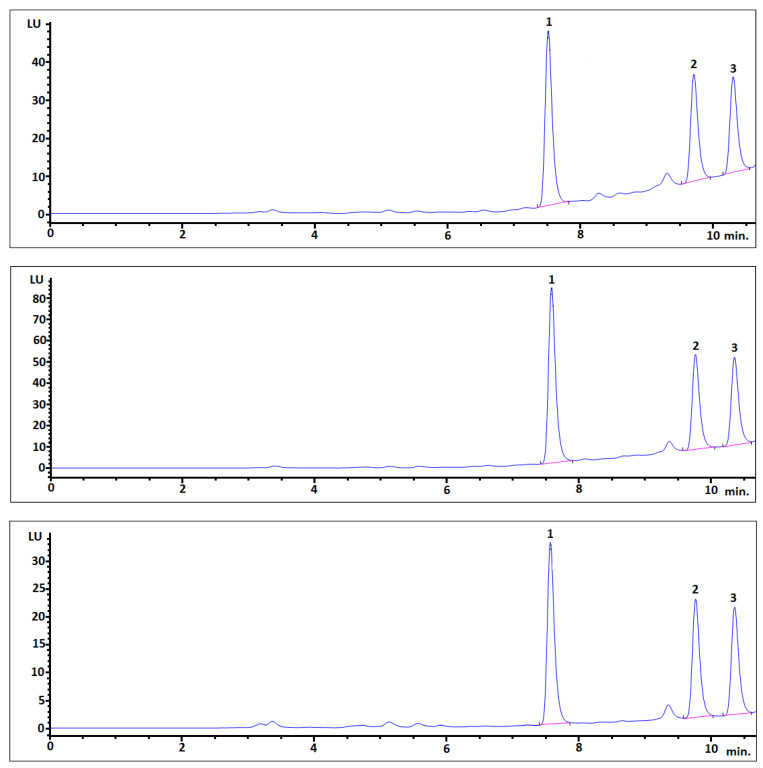
Comparison of the enrichment levels of urine human samples at three following levels (from top to below): 1 × LOQ (a reinforcement—10), 1.5 × LOQ, and 3 × LOQ (a reinforcement—12 for top and middle, a reinforcement—10 for bottom).

Chromatograms from spiked samples to the average blank human urine matrix samples at three levels (100, 150, and 300 nanograms per milliliter of sample) after SPE described in **Materials and Methods** (Section 2.7*. SPE-based extraction procedure*). Majority of analytes are well separated from remaining matrix coextractives (Figure 1). Average analyte recovery from spiked samples for the d-SPE before SPE procedure were satisfactory for three bisphenols such as BADGE 2H_2_O, BPF, and bisphenol E (BPE) are 81, 73, and 72%, respectively. The d-SPE/SPE gives similar or slightly better results in terms of matrix effects and recovery rates in addition to offering higher sensitivity; it would probably be the better option when HPLC-FLD will be used in other biological samples.

Recovery studies conducted at three spiking levels of 100, 150, and 300 ng/mL proved that the elaborated extraction procedures, especially SPE, support the possibility of bisphenol residue determination in human urine samples. 

Detailed validation data of SPE procedure are provided in Table 4. Satisfactory recovery, intra- and inter-day repeatability, and intra-laboratory reproducibility were obtained for spiked urine samples (Table 4) [44]. 

Intra-day accuracy (Recovery %) and precision relative standard deviation (RSD%) at the following three levels were calculated: 1 × LOQ, 1.5 × LOQ, and 3 × LOQ. On the basis of all 54 experiments (18 replicates analyzed on three different days for three different LOQs levels), inter-day repeatability was calculated obtaining recovery in the range 73.7–86.8% with RSD% values in the range 3.4–7.9%. As for intra-laboratory reproducibility of all 90 experiments (experiments conducted by two different analysts, 36 replicates analyzed on three different days for three different LOQs levels), recoveries were in the range 73.8–86.8% with RSD% values in the range 2.5–8.2%. 

In total, 90 different samples were analyzed with overall accuracy and precision in the range 73.7–86.8% and 3.8–7.4%, respectively (Table 4). 

The conditions of the chromatographic analysis and the parameters of the FLD detector were optimized. Sufficient sensitivity HPLC-FLD was achieved applying reinforcements of described studies to analytes (10 or 12 from range 1–18). The optimized conditions of analyses allow for the selective enhancement of analytes in urine samples, which are separated from the remaining matrix interferences. The HPLC-FLD separation of all compounds in 11 min is very effective because all the analytes are resolved and can be quantified; moreover, very low LOQs were attained in the range 11.42–22.35 ng mL^−1^ (Table 3). 

The method limits of detection (mLODs) and method limits of quantification (mLOQs) values of all bisphenols were in the range 18.00–33.90 ng mL^−1^ and in the range 54.53–102.73 ng mL^−1^, respectively (Table 3). During the HPLC-FLD experiments a moderate reinforcement of studies analytes (10) from the applicable reinforcement range (1–18) was used. Obviously, when using the highest analytes reinforcement (18), their mLOD and mLOQ values were much lower.

**Figure 2:**
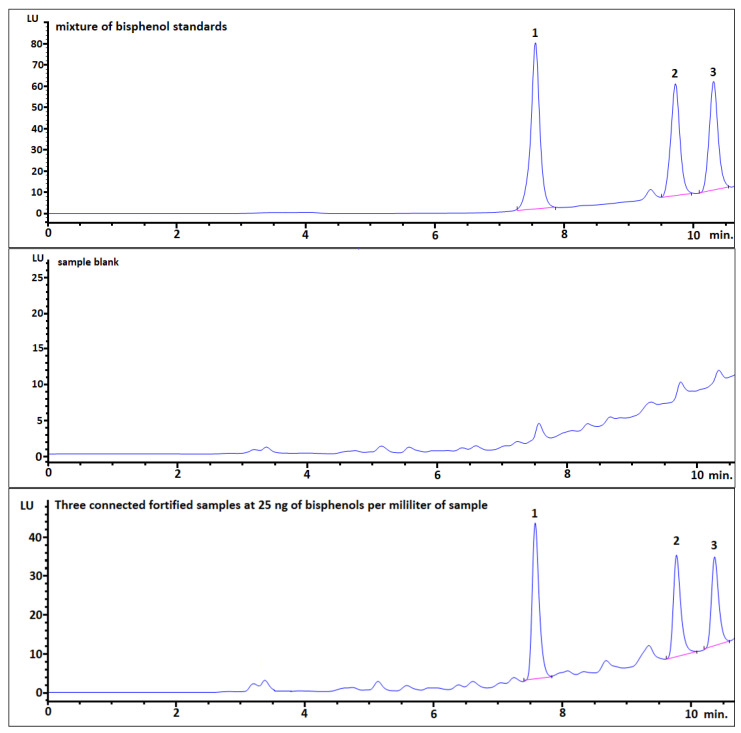
Comparison of following chromatograms: mixture of bisphenol standards (**top**); chromatogram of blank human urine matrix sample (**middle**); three samples of blank human urine matrix samples which were spiked with mixture of bisphenol standards at the same level (25 ng/mL), after the SPE procedure, then were evaporated to dryness, connected, and reconstituted as one sample for analysis (**bottom**).

The validated method was applied to the analysis of bisphenols in human urine samples from volunteers living in Lublin, Poland. During the quantification of bisphenols in urine samples, individual eluates were evaporated separately. As shown in Figure 2, after evaporated to dryness, extracts were then connected and reconstituted as one sample (300 µL MeCN: water, 30: 70 (*v/v*) (3 × 100 µL)).

In the first twelve urine samples of 26-year-old men, concentrations of bisphenols close to the LOQs were detected. BADGE∙2H_2_O was detected in 11 samples (92%); BPF and BPE were detected in seven samples (58%) All three analyzed BPs were detected simultaneously in six samples. A pair of BPs was detected in one sample. Detailed results are shown in Table 1.

## 4. Conclusions

The subject of this paper was the optimization of the conditions for the identification and quantitative analysis of bisphenols by HPLC-FLD and the optimization of the extraction conditions of analytes in urine samples using the SPE technique with Strata Phenyl SPE columns. The Strata Phenyl SPE columns with short alkyl chain with a phenyl group provides moderate hydrophobic selectivity and aromatic selectivity through π–π interactions and ensures high recovery values of bisphenols.

The optimized procedure enables the identification of analytes in urine samples with a very small volume of 500 µL. The ability to identify and quantify bisphenols in a volume of 500 µL of urine is particularly important when collecting urine samples from children, whose volumes are smaller compared to urine samples collected from adults. Therefore, the optimized procedure for the analysis of bisphenols in small volumes of urine can be successfully used in the analysis of children’s urine samples.

During the bisphenols HPLC-FLD analysis, from 6 min reinforcements of 10 or 12 were used, and analytes were identified. Optimizing the conditions of the chromatographic analysis, the parameters of the FLD detector, including the use of an appropriate, optimal amplification of the analytes (10 or 12) from the sixth minute of the chromatographic process, allows for the selective enhancement of analytes in urine samples, which are separated from the remaining matrix components that mostly elute at first.

Intra-day and inter-day precision of the method was good, with relative standard deviation (% RSD) ≤ 8.2%.

From the total twelve human urine samples, in the six samples of human urine, three bisphenols below the LOQs values were identified. BADGE × 2H_2_O was detected in the majority of human urine samples at concentration range LOD-LOQ (at ≤15 ng/mL), while BPF and BPE were identified rarely (7 ng/mL to LOQs).

Application of HPLC-FLD allowed quantification of bisphenols in a total twelve human urine samples. The proposed SPE (or d-SPE/SPE) procedure could be recommended for further effective and reliable selected bisphenols analysis in small amount human urine samples, including children.

## Figures and Tables

**Table 1:** Results of determination BPs in 12 different human urine samples.

Sample Number	Determined Bisphenols		
	BADGE∙2H_2_O	BPF	BPE
1	<LOQ	<LOQ	<LOQ
2	<LOQ		
3	<LOQ	<LOQ	<LOQ
4	<LOQ	<LOQ	<LOQ
5		<LOQ	<LOQ
6	<LOQ	<LOQ	<LOQ
7	<LOQ		
8	<LOQ		
9	<LOQ		
10	<LOQ		
11	<LOQ	<LOQ	<LOQ
12	<LOQ	<LOQ	<LOQ

**Table 2:** Physicochemical properties of the selected bisphenols.

No.	Bisphenol	IUPAC Name	Chemical Structure	Molecular Weight ^1^ (g/mol)	Log *P* ^1^	Proton Donors ^1^	Proton Acceptors ^1^
1	**BADGE∙2H_2_O**	2,2-bis[4 -(2,3-hydroxypropoxy)phenyl]propane	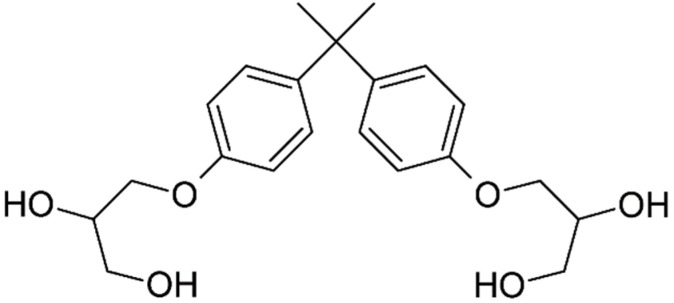	376.4	2.1	4	6
2	**BPF**	4-[(4-hydroxyphenyl)methyl]phenol	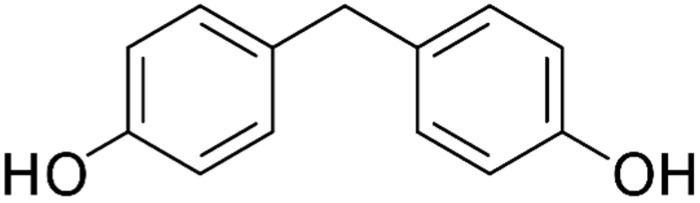	200.23	2.9	2	2
3	**BPE**	4-[1-(4-hydroxyphenyl)ethyl]phenol	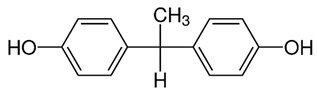	214.26	3.9	2	2

^1^—data obtained from PubChem database.

**Table 3:** Validation parameters for the method: retention times, calibration curves equations (which were constructed using methanol), correlation coefficients (*r*), limits of detection (LODs), and limits of quantification (LOQs) obtained for the three bisphenols by HPLC-FLD. Validation parameters for the method: method limits of detection (mLODs) and method limits of quantification (mLOQs) obtained for the three bisphenols by HPLC-FLD method after SPE of the human urine samples.

No.	Bisphenol	Retention Time, *t*r (min)	Concentration Range (ng mL^−1^)	λ (nm)	Linear Regression	Correlation Coefficient (*r*)	LOD (ng mL ^−1^)	LOQ (ng mL ^−1^)	mLOD (ng mL ^−1^)	mLOQ (ng mL ^−1^)
**1**	BADGE∙2H_2_O	~7.5	10–500	240	y = 1.5781x + 9.9644	*r* = 0.9995	7.37	22.35	33.90	102.73
**2**	BPF	~9.7	10–500	240	y = 0.9516x + 2.286	*r* = 0.9999	3.77	11.42	18.93	57.35
**3**	BPE	~10.3	10–500	240	y = 0.7022x − 2.5784	*r* = 0.9999	4.42	13.39	18.00	54.53

**Table 4:** Mean recoveries (%) and relative standard deviations expressed as a percentage (RSD%) for mixture of bisphenols extracted by SPE using Strata Phenyl SPE column.

Recoveries Obtained for Fortification at 100 ng/mL (1 × LOQ) Sample after the SPE Procedure																
Bisphenol	Intra-Day Repeatability ^a^						Inter-Day Repeatability ^b^ (n = 18)		Intra-Laboratory Reproducibility ^c^						Overall ^d^ (n = 30)	
Name	Day 1 (n = 6)		Day 2 (n = 6)		Day 3 (n = 6)				Analyst 1 (n = 6)		Analyst 2 (n = 6)		Mean (n = 12)			
	Recovery %	RSD%	Recovery %	RSD%	Recovery (%)	RSD%	Recovery %	RSD%	Recovery %	RSD%	Recovery %	RSD%	Recovery %	RSD%	Recovery %	RSD%
**BADGE·2H_2_O**	85.4	5%	84.8	2%	84.7	4%	85.0	3.7%	85.0	4%	84.8	4%	84.9	4.0%	85.0	3.8%
**BPF**	76.5	6%	75.5	5%	76.5	3%	76.2	4.8%	75.8	5%	75.8	11%	75.8	8.2%	76.0	6.1%
**BPE**	74.0	3%	73.8	4%	74.0	7%	73.9	4.9%	73.7	5%	74.0	7%	73.8	5.9%	73.9	5.3%
**Recoveries Obtained for Fortification at 150 ng/mL (1.5 × LOQ) Sample after the SPE Procedure**																
**Bisphenol**	**Intra-Day Repeatability ^a^**						**Inter-Day Repeatability ^b^** **(n = 18)**		**Intra-Laboratory Reproducibility ^c^**						**Overall ^d^** **(n = 30)**	
**Name**	**Day 1 (n = 6)**		**Day 2 (n = 6)**		**Day 3 (n = 6)**				**Analyst 1 (n = 6)**		**Analyst 2 (n = 6)**		**Mean (n = 12)**			
	**Recovery %**	**RSD%**	**Recovery %**	**RSD%**	**Recovery (%)**	**RSD%**	**Recovery %**	**RSD%**	**Recovery %**	**RSD%**	**Recovery %**	**RSD%**	**Recovery %**	**RSD%**	**Recovery %**	**RSD%**
**BADGE·2H_2_O**	86.5	9%	87.0	1%	86.8	5%	86.8	5.2%	86.3	1%	87.2	5%	86.8	3.0%	86.8	4.3%
**BPF**	76.0	10%	75.7	3%	76.5	4%	76.1	5.6%	76.3	6%	76.0	6%	76.2	6.0%	76.1	5.7%
**BPE**	73.8	8%	73.5	7%	73.7	9%	73.7	7.9%	73.3	7%	74.2	7%	73.8	6.6%	73.7	7.4%
**Recoveries Obtained for Fortification at 300 ng/mL (3 × LOQ) Sample after the SPE Procedure**																
**Bisphenol**	**Intra-Day Repeatability ^a^**						**Inter-Day Repeatability ^b^** **(n = 18)**		**Intra-Laboratory Reproducibility ^c^**						**Overall ^d^** **(n = 30)**	
**Name**	**Day 1 (n = 6)**		**Day 2 (n = 6)**		**Day 3 (n = 6)**				**Analyst 1 (n = 6)**		**Analyst 2 (n = 6)**		**Mean (n = 12)**			
	**Recovery %**	**RSD%**	**Recovery %**	**RSD%**	**Recovery (%)**	**RSD%**	**Recovery %**	**RSD%**	**Recovery %**	**RSD%**	**Recovery %**	**RSD%**	**Recovery %**	**RSD%**	**Recovery %**	**RSD%**
**BADGE·2H_2_O**	84.6	2%	84.2	3%	84.2	5%	84.3	3.4%	84.5	2%	84.2	3%	84.3	2.5%	84.3	3.0%
**BPF**	76.5	5%	76.2	5%	76.3	3%	76.3	4.2%	75.7	3%	76.0	5%	75.8	4.1%	76.1	4.2%
**BPE**	74.2	4%	74.3	5%	74.2	4%	74.2	4.3%	74.7	4%	73.8	3%	74.3	3.2%	74.2	3.9%

**^a^**—mean recovery % and RSD% for within-day results of batch of six samples per day (n = 6). **^b^**—mean recovery % and RSD% from 18 samples analyzed in three different days (n = 6 for each day). **^c^**—mean recovery % and RSD% from experiments conducted by two different analysts (n = 6 for each operator) and average results (n = 12). **^d^**—average recovery % and RSD% from all experiments (n = 30).

## Data Availability

Data is contained within the article.

## Sample Availability

Samples of the compounds are not available.
